# Whole genome sequencing of simmental cattle for SNP and CNV discovery

**DOI:** 10.1186/s12864-023-09248-x

**Published:** 2023-04-05

**Authors:** Ting Sun, Shengwei Pei, Yangkai Liu, Quratulain Hanif, Haiyue Xu, Ningbo Chen, Chuzhao Lei, Xiangpeng Yue

**Affiliations:** 1 State Key Laboratory of Herbage Improvement and Grassland Agro-ecosystems, Key Laboratory of Grassland Livestock Industry Innovation, Ministry of Agriculture and Rural Affairs, Engineering Research Center of Grassland Industry, College of Pastoral Agriculture Science and Technology, Ministry of Education, Lanzhou University, Lanzhou, 730020 P. R. China; 2 Key Laboratory of Animal Genetics, Breeding and Reproduction of Shaanxi Province, College of Animal Science and Technology, Northwest A&F University, Yangling, 712100 Shaanxi China; 3 College of Life Sciences, Shaanxi Normal University, Xi’an, 710062 China; 4 Computational Biology Laboratory, Agricultural Biotechnology Division, National Institute for Biotechnology and Genetic Engineering, Faisalabad, Pakistan; 5 Department of Biotechnology, Pakistan Institute of Engineering and Applied Sciences, Nilore, Islamabad, Pakistan

**Keywords:** Simmental cattle, Selection, Sperm motility, SNP, CNV

## Abstract

**Backgroud:**

The single nucleotide polymorphisms (SNPs) and copy number variations (CNVs) are two major genomic variants, which play crucial roles in evolutionary and phenotypic diversity.

**Results:**

In this study, we performed a comprehensive analysis to explore the genetic variations (SNPs and CNVs) of high sperm motility (HSM) and poor sperm motility (PSM) Simmental bulls using the high-coverage (25×) short-read next generation sequencing and single-molecule long reads sequencing data. A total of ~ 15 million SNPs and 2,944 CNV regions (CNVRs) were detected in Simmental bulls, and a set of positive selected genes (PSGs) and CNVRs were found to be overlapped with quantitative trait loci (QTLs) involving immunity, muscle development, reproduction, etc. In addition, we detected two new variants in *LEPR*, which may be related to the artificial breeding to improve important economic traits. Moreover, a set of genes and pathways functionally related to male fertility were identified. Remarkably, a CNV on *SPAG16* (chr2:101,427,468 − 101,429,883) was completely deleted in all poor sperm motility (PSM) bulls and half of the bulls in high sperm motility (HSM), which may play a crucial role in the bull-fertility.

**Conclusions:**

In conclusion, this study provides a valuable genetic variation resource for the cattle breeding and selection programs.

**Supplementary Information:**

The online version contains supplementary material available at 10.1186/s12864-023-09248-x.

## Backgroud

Cattle were domesticated around 10,000 years before present (YBP), providing mankind with meat, milk, skin, and working power, etc. The natural and artificial selection has left phenomenal stress marks on the cattle genome determining its phenotype, adaptation and production performance. To date, a large number of studies have been reported in various cattle populations based on the genomic single nucleotide polymorphisms (SNPs), and a set of candidate genes were identified to be related to reproduction, meat, milk and environmental adaption. For instance, three genes (*MATR3*, *MZB1* and *STING1*) are related to host immune, and *SOD1*, *PRLH* and *DNAJC18* genes are associated with environmental thermal stress in the African cattle [1, 2]. Significantly, hundreds of candidate regions under positive selection among different cattle breeds were detected, which were responsible for production, growth, reproduction, immune response and milk production [3, 4].

Copy number variation (CNV) is another kind of genomic variant, ranging from 50 bp to 5 Mb [5]. Compared to the SNPs, CNV has a greater influence on the function, phenotype and evolution by the gene dosage, coding sequence, and regulation of long-range genes [6, 7]. The CNV in many species has been investigated based on comparative genomic hybridization arrays (CGH array), SNP arrays (Illumina BovineHD BeadChip, Illumina BovineSNP50 BeadChip), short-read next-generation sequencing (NGS) and single-molecule long-read sequencing (SMRT) methods. Compared to array technologies, NGS and SMRT exhibit higher precise breakpoints, sensitivity and resolution [8, 9]. As for the short-reads NGS data, many detection methods have been developed according to four strategies: read pair (RP), split read (SR), read depth (RD) and genome-based assembly (AS) [10]. Each strategy has its own strengths and weaknesses, and none of them can detect all types of CNVs. The SMRT can substantially improve the reliability and resolution of variant detection [11]. However, due to the high cost, limited studies were conducted to detect CNV in cattle genome using SMRT.

The Simmental cattle, a beef/milk dual-purpose breed, is one of the most widely distributed cattle breeds in the world. Previous studies have explored the selective signatures and copy number variation in Simmental cattle using different SNP arrays. The selection signature was firstly investigated in a large population of Simmental cattle using Illumina BovineSNP50, which identified 224 candidate regions containing genes associated with important economical traits [12]. Another study identified 263 CNV regions (CNVRs) in the genome of Simmental cattle using Illumina Bovine HD BeadChip, revealing that genes in CNVRs are related to transmembrane activity and olfactory transduction activity [13]. Afterward, various genome-wide association studies (GWAS) have been performed to identify the candidate genes/loci associated with economic traits of Simmental cattle, including carcass, meat, and growth [14–16]. Although it is feasible to detect the positive selective signatures and CNV using the SNP array, the limited resolution reduces the sensitivity and accuracy of detection. In addition, fertility plays an important role in the success of calf production. To date, most studies primarily focused on the female fertility, while male fertility has received much less attention. Though previous studies have identified a set of single-nucleotide polymorphisms (SNPs) associated with bull fertility based on the SNP array [16–19], the effective markers are still lacking for elite bull selection.

In the current study, combining the high-coverage short-read NGS data and SMRT data, a comprehensive analysis was conducted to identify the genetic variations (SNPs and CNVs) in the genome of Simmental bulls, which identified a set of positive selected genes (PSGs) and CNVRs overlapped with quantitative trait loci (QTLs) involving in milk, immunity, reproduction. Significantly, a CNV on *SPAG16* was completely deleted in all poor sperm motility (PSM) bulls and half of the high sperm motility (HSM) bulls, indicating its important role in bull fertility.

## Results

### Genomic landscape of SNPs and CNVs in Simmental bulls

In the present study, 30 Simmental cattle were sequenced generating ~ 2.17 billion paired-end reads with the average coverage depth of ~ 25×. The reads were aligned to the high-quality taurine reference genome (ARS-UCD1.2) [20] with an average alignment rate of 99.83% (Table S1), generating 15,154,539 autosomal SNPs (121,568 with a minor allele frequency < 1%, 2,643,818 between 1% and 5%, and 11,490,159 > 5%) which were used for further downstream analysis.

**Fig. 1:**
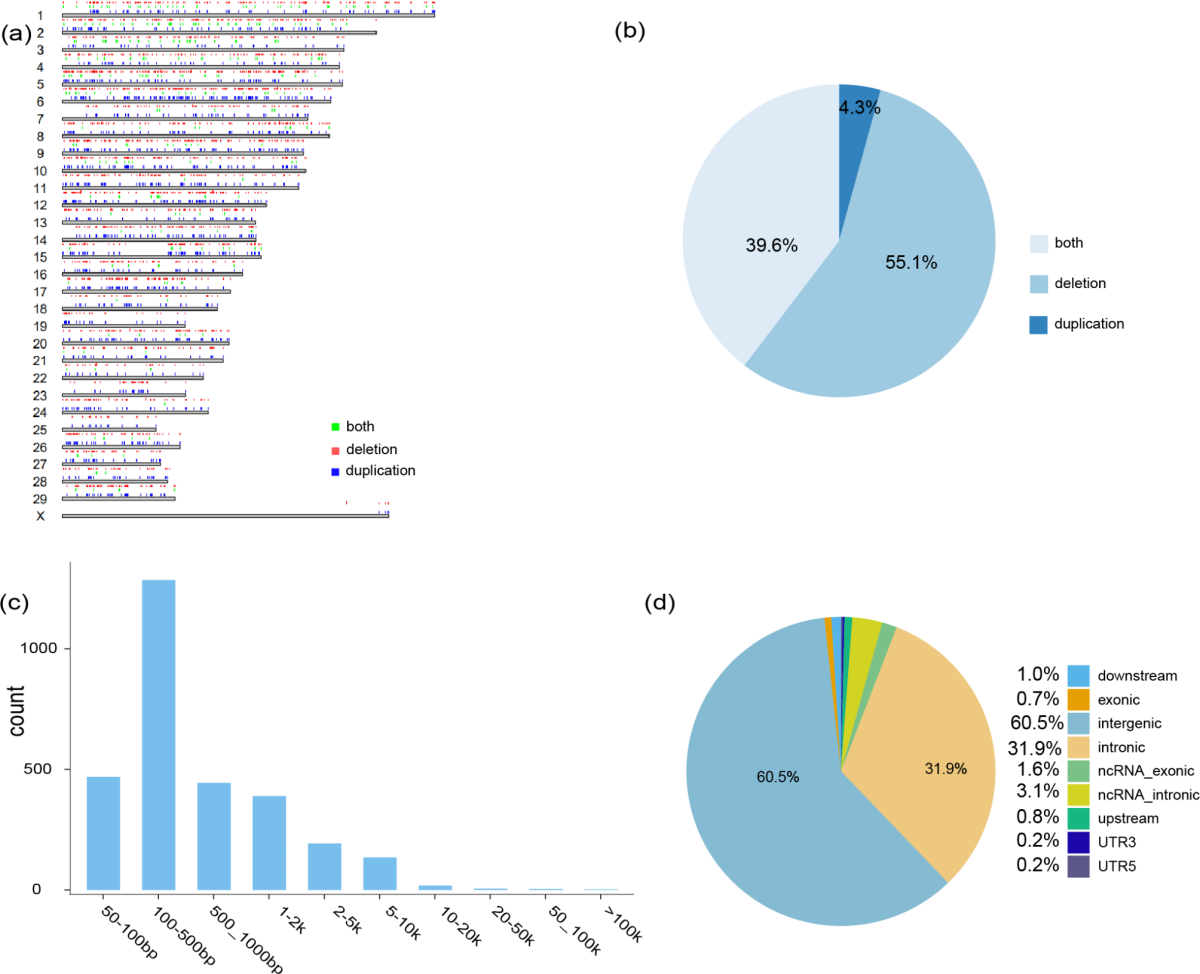
(a) Genomic distribution of CNVRs. Green: both, red: deletion, blue: duplication. (b) Frequency of different types of CNVR (c) CNVR length distribution. (d) Functional classification of the detected CNVRs

In addition, we constructed a confidential CNV dataset using high-throughput Nanopore long-reads (PromethION) and the high coverage Illumina short read sequencing data. A total of 2,944 copy number variant regions (CNVRs) were detected (Fig. 1a &c, Table S2), including 1,651 deletions, 126 duplications, 1,167 both events, with a total length of 4,661,581 bp and an average length of 1,583 bp, covering 0.18% of the genome. The length of CNVRs was found to be mainly distributed within 100–500 bp, accounting for ~ 43.65% of the detected CNVR (Fig. 1b, Table S3). Moreover, with the increase in its length (> 500 bp), the number of CNVRs decreases. In addition, our results showed that CNVRs were not uniformly distributed across the genome, of which ~ 60.53% of CNVRs (1,782) were located in the intergenic region, only 0.71% in the exon region (Fig. 1d). Moreover, there were 11 CNVRs overlapped with the QTLs related to immunity, milk and production traits (Table S4).

### Selection signature analysis for Simmental cattle based on autosomal SNPs

Three statistical methods (Pi, CLR, and iHS) (Table S5-S7) were applied to explore the positive selection signatures in Simmental cattle. For each method, regions showing outlier values (top 1%) were selected as the candidate genomic regions (Figure S2a). The positive selected genes (PSGs) were identified by at least two approaches, and a total of 235 PSGs were identified.

Totally, 53 PSGs were intersected with cattle QTLs (containing 476 QTLs) [21], which were associated with immunity, meat, milk, production, and reproduction (Table S8). For example, a region on chromosome 16 containing *RERE* and its neighboring genes (*LOC112441839* and *SLC45A1*) was observed with markedly higher values, falling in 4 QTLs associated with reproduction (Figure S2 a&b, Table S8). In addition, another ~ 2.5 Mb region on chromosome 7 also showed markedly higher values, containing ten genes (*ANKHD1*, *CDC23*, *CXXC5*, *CYSTM1*, *FAM13B*, *KDM3B*, *LOC101904825*, *NRG2*, *PSD2*, *PURA*), overlapped with QTLs associated with immunity, milk and reproduction in cattle (Figure S2 a&c, Table S8).

**Fig. 2:**
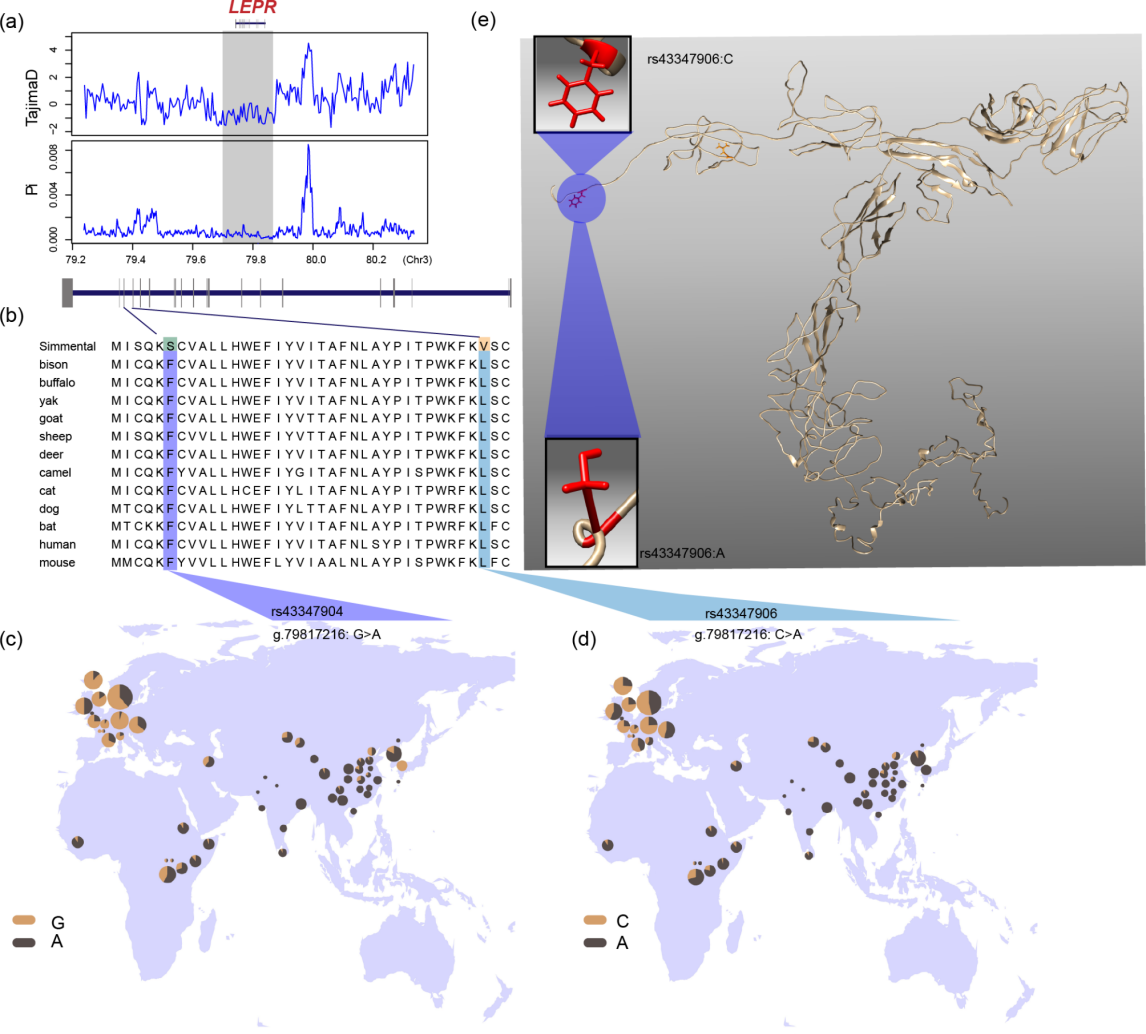
Selection and conservative analysis of *LEPR*. (a) Selective sweep detected by Tajima’s D and Pi test. (b) conservative analysis of two loci. (c & d) Geographical distribution of the two variants (http://animal.nwsuaf.edu.cn/code/index.php/BosVar) (e) 3D protein structure prediction of *LEPR*

Moreover, KOBAS [22] was used to perform GO and KEGG pathway analysis based on the 235 PSGs (Table S9-S10). The KEGG pathways resulted in two significantly over-represented pathways: cytokine-cytokine receptor interaction (corrected P-Value = 0.0159) and Oocyte meiosis (corrected P-Value = 0.0177). A total of 8 genes (*TNFSF13*, *TNFSF12*, *LEPR*, *IFNAR2*, *EDAR*, *CX3CL1*, *GDF15*, *CCL22*) were involved in the cytokine-cytokine receptor interaction pathway. Notably, two conserved nonsynonymous mutations (rs43347904, g.79,817,216: G > A, exon3, p.S6F; rs43347906, g.79,817,216: C > A, exon4, p.V35L) were detected within the *LEPR* gene (Fig. 2). *LEPR* was detected within a QTL related to reproduction in cattle [21]. We further investigated the frequency of these two mutations across the diverse cattle breeds around the world using the Bovine Genome Variation Database and Selective Signatures (BGVD, http://animal.nwsuaf.edu.cn/code/index.php/BosVar) (Fig. 2c and d). The allele G of rs43347904 showed a high frequency in European (0.724) and Eurasian (0.789) cattle populations. In Simmental cattle populations, the allele G showed a higher frequency of 0.957. It was also observed in some Chinese and African cattle breeds with a low frequency. The rs43347906 showed a similar pattern, and alters the protein structure (Fig. 2e). In addition, by investigating published literatures, we found a set of genes associated with immunity (*EGR1*, *MUC6*), and muscle development (*MEIS1*, *GDF15*) (Table 1).

**Table 1:** Summary of Partial Traits Associated with Positively Selected Genes

Gene	Trait	Reference
*GDF15*	skeletal muscle growth; body weight	[47, 48]
*LEPR*	Reproductive Traits	[51, 52, 55, 81, 82]
*MUC6*	immunity	[83, 84]
*MEIS1*	muscle	[85]
*EGR1*	immunity	[86]

### Identification of candidate genes/CNVRs associated with fertility

Sperm motility is one of the major determinants of male fertility. According to the sperm motility, the 30 Simmental bulls were divided into two groups: the HSM (n = 14) and the PSM group (n = 16). For HSM group, the sperm motility of fresh semen and frozen-thawed semen were 0.68 ± 0.04, and 0.36 ± 0.02, respectively, while were 0.32 ± 0.11 and 0.15 ± 0.05 for PSM group, respectively. To investigate the group-specific selection between HSM and PSM groups, we scan for genomic regions from genome-wide SNP and CNV datasets, respectively.

Firstly, we used the *F*_ST_ to scan genomic regions with extreme allele frequency differentiation between HSM and PSM groups using the SNP dataset. Using the top 1% of *F*_ST_ values, we identified 564 candidate selective regions covering 599 genes. To obtain a broad overview of the molecular functions of these candidate genes, we performed GO and KEGG enrichment analysis using KOBAS [22]. We detected three significantly over-represented (corrected *P*-value < 0.05) KEGG pathways related to the male fertility (insulin secretion (corrected *P*-value = 0.01537), oxytocin signaling pathway (corrected *P*-value = 0.016431), calcium signaling pathway (corrected *P*-value = 0.02558)) (Table S11). Besides, we identified 13 significantly over-represented GO terms (Table S12), such as potassium ion transmembrane transport, stabilization of membrane potential, potassium ion leak channel activity, calcium ion binding, etc.

In addition, a comparison of these detected candidate regions and known QTLs revealed that these detected candidate genes are overlapped with cattle QTLs associated with reproduction, production, and health (Table S13). Totally, our results showed that there were 290 candidate genes were observed to overlap with 2,611 cattle QTLs (Table S13). Among these 2,611 cattle QTLs, 284 QTLs covering 101 genes (such as *SERPINE2*, *AGBL4*, *SORCS1*, *TMEM181*, *SPAG16*) were associated with reproduction traits, such as sperm motility (*AGBL4*, *SORCS1*, *SPAG16*), sperm concentration (*TMEM181*), and fertilization rate (*SERPINE2*).

**Fig. 3:**
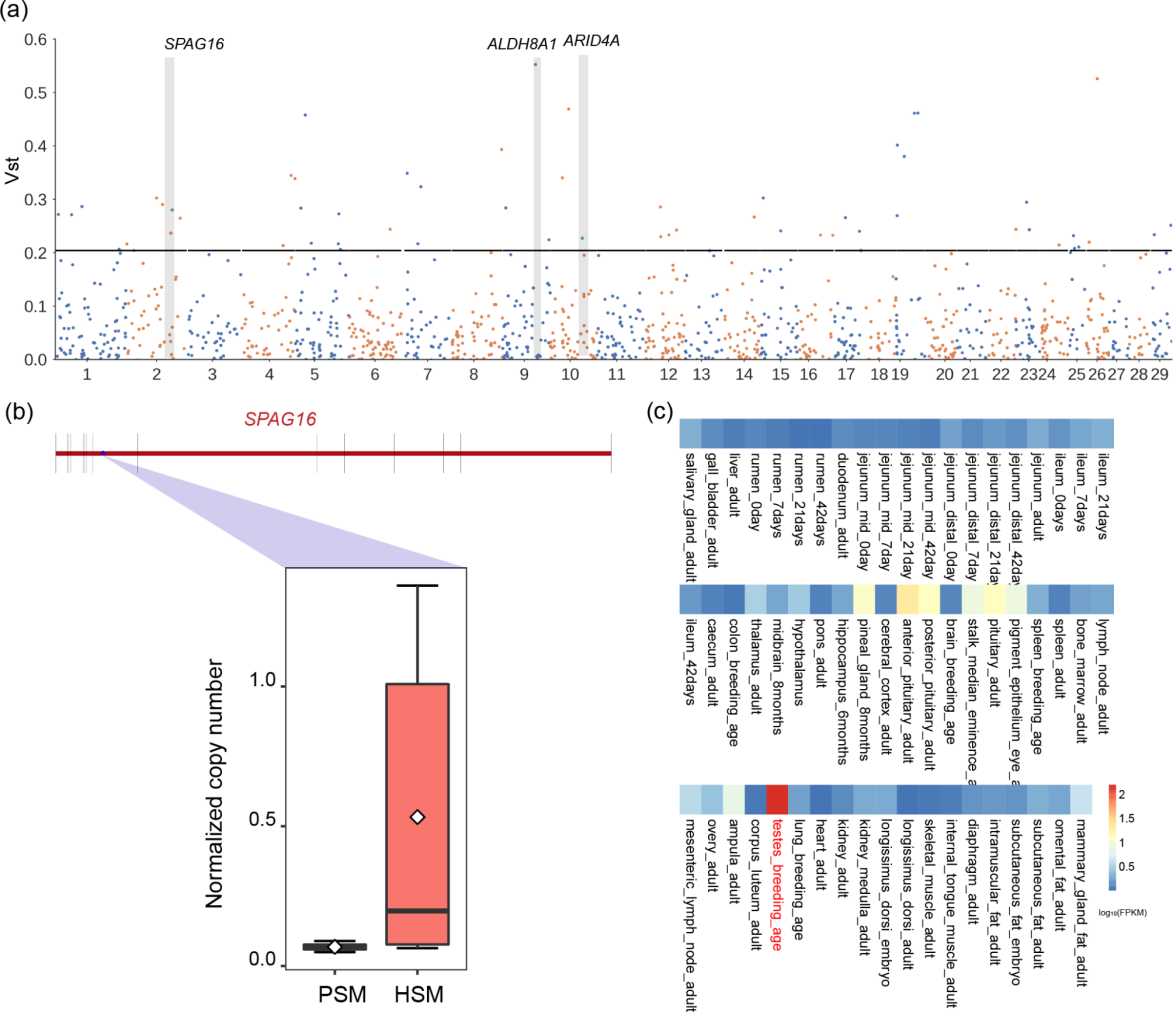
(a) Manhattan plot for V_ST_ (top 2%). (b) Boxplot of normalized copy number of *SPAG16*. (c) Gene expression of *SPAG16* in different cattle tissues (http://animal.nwsuaf.edu.cn/code/index.php/RGD)

Moreover, we identified 58 highly differentiated CNVRs (top 2% of V_ST_ value) between the two groups (Fig. 3a) by calculating V_ST_ based on the confidential CNV dataset constructed in this study. Our results showed that 26 CNVRs were found to be located in the intergenic region, while 21 lay in the intronic region, overlapping with 31 genes. Some of these genes (such as *ARID4A*, *ALDH8A1*, and *SPAG16*) were involved in the male fertility. We detected a significantly differential deletion (chr2:101427468–101,429,883) covering the intronic region of *SPAG16* gene (Fig. 3b). We used the PCR to check the existence of this CNV segment in 30 Simmental cattle (Figure S3). The results showed that this region was a complete deletion in all the PSM bulls and half of the HSM bulls based on the genotyping information, which confirmed our observation in the genome sequencing analysis. In addition, the *SPAG16* was also identified as a candidate gene in the *F*_ST_. Moreover, the expression level of *SPAG16* in cattle was significantly higher in testis than in other tissues (http://animal.nwsuaf.edu.cn/code/index.php/RGD/loadByGet?address[]=RGD/Items/ExprCattle), indicating that *SPAG16* may play an important role in the male fertility of Simmental cattle (Fig. 3c).

## Discussion

As one of the most important and widely distributed cattle breeds, Simmental cattle are mainly used for milk and beef purposes. In the current study, we detected 15,154,539 autosomal SNPs in Simmental cattle. Besides, we firstly constructed a confidential CNV data set for Simmental cattle using different sequencing platforms (NGS and long-reads), multi-strategies, and the newly reported high-quality genome (ARS-UCD1.2) [20], which ensure us to obtain a highly confidential CNV dataset. Totally, we detected a total of 2,944 CNVRs for 30 Simmental cattle, which showed a similar level to other the cattle breeds [23]. Compared with cattle QTL database, there were 11 CNVRs overlapped with QTLs related to immunity, milk and production traits [21], indicating that CNV may be a critical type of genetic variation, may have an important effect on cattle fertility, health, and economic traits.

Over the last few decades, strong human driven selection contributed immensely to productive traits enhancement within the Simmental cattle genome. It is worthwhile to identify the candidate gene during the domestication, which will accelerate the improvement of important traits of cattle in the future. In this study, we used three methods (Pi, CLR, and iHS) to improve the power of detecting selection signatures [24], and a total of 235 PSGs were identified for Simmental cattle. To further explore the hereditary effects, the detected PSGs were compared with cattle QTLs [21]. A total of 53 PSGs overlapped with 469 QTLs related to immunity, meat, milk, production, and reproduction [21] (Table S1, Table S2). Notably, a ~ 2.5 Mb region on chromosome 7 containing several genes (*ANKHD1*, *CDC23*, *CXXC5*, *CYSTM1*, *FAM13B*, *KDM3B*, *LOC101904825*, *NRG2*, *PSD2*, *PURA*) and another region on chromosome 16 containing *RERE* gene and its neighboring genes (*LOC112441839* and *SLC45A1*) showing high values, was overlapped with QTLs associated with milk and reproduction traits in cattle [21]. Studies showed that *CYSTM1* and *NRG2* are significantly related to the gestation length [25, 26]. *KDM3B*, a histone H3 demethylase, plays a crucial role in spermatogenesis and normal male sexual behavior, which is also identified as a fertility-related candidate gene for sheep [27]. *CXXC5* and *PSD2* were involved in the function of fat deposition [28, 29]. The *RERE* has been identified as a candidate gene associated with reproductive development [30–32]. The functional analysis (KEGG pathway and GO) performed based on the 235 PSGs showed that cytokine-cytokine receptor interaction (corrected P-Value = 0.0159) and oocyte meiosis (corrected P-Value = 0.0177) were significantly over-represented. The oocyte meiosis pathway has been reported to be important in reproduction [33, 34]. The cytokine-cytokine receptor interaction pathway plays a central role in immunity, which is also related to backfat thickness [35], reproduction [34], growth of the animal [36], feed conversion ratio in beef cattle [37], beef quality [38], and meat production [39]. Moreover, previous studies indicated that this pathway plays an important role in the natural and artificial selection in the process of sheep domestication [40, 41]. There are several genes (*TNFSF12*, *TNFSF13*, *IFNAR2*, *EDAR*, *CX3CL1*, *GDF15*, *CCL22*, *LEPR*) involved in cytokine-cytokine receptor interaction pathway. *TNFSF12* and *TNFSF13*, belonging to the tumor necrosis factor (TNF) ligand superfamily, involved in many cellular activities, play an important role in the immunological responses in animals [42, 43]. *CX3CL1*, a member of chemokine repertoire, is related to immune-related inflammatory diseases in humans [44, 45]. *CCL22* is a cytokine gene, which plays an important role in immunity [46]. Tsai et al. showed that *GDF15* can regulate the appetite and body weight [47], while Gurgul et al. identified *GDF15* as a candidate gene related to the skeletal muscle growth in cattle [48]. *LEPR*, a member of class I cytokine receptor superfamily, encodes leptin receptor. Studies have been revealed that leptin acts via leptin receptor, regulating the satiety and fat deposition [49, 50]. To date, *LEPR* has been widely reported to be related to meat, milk, reproduction, and growth traits in cattle [51–55]. We detected two non-synonymous mutations (rs43347904, g.79,817,216: G > A, exon3, p.S6F; rs43347906, g.79,817,216: C > A, exon4, p.V35L) within the *LEPR* gene. The two mutations (G of rs43347904, C of rs43347906) exhibited a high frequency in European and Eurasian cattle population (especially in the Simmental breed), while almost absent in African taurine, Indian and Chinese indicine. In addition, both of these variants were also present in some Chinese and African indicine breeds with a low frequency, which might be due to the hybridization of *Bos taurus* and *Bos indicus*. Interestingly, our results suggested that these two variants showed significantly high frequency in the cattle breed (such as Holstein cattle, Angus cattle, Hanwoo cattle, Mishima cattle, etc.) with good economic traits. The G for rs43347904 and C for rs43347906 were conserved across other mammal sequences (Fig. 2b). Combining the conservation and allele distribution pattern of these two variants, we speculated that these (C of rs43347906 and G of rs43347904) alleles originated from *Bos taurus* and may be related to the artificial breeding to improve the economic traits.

Furthermore, we divided the 30 Simmental cattle into HSM and PSM groups for selective sweep analysis to identify candidate genes/CNVR associated with sperm motility using the genome-wide SNP and CNV dataset, respectively. The *F*_ST_ was calculated to scan for genomic regions with extreme allele frequency differentiation between HSM and PSM cattle using the SNP dataset. Totally, we identified 599 candidate genes, and the further enrichment analysis for these genes showed that insulin secretion, oxytocin signaling pathway, and calcium signaling pathway were significantly over-represented. Studies showed that insulin plays an important role in sperm capacitation and spermatogenesis. Oxytocin can stimulate contractions of the reproductive tract to help sperm release [56]. In addition, our results showed that 101 genes (e.g., *SERPINE2*, *AGBL4*, *SORCS1*, *TMEM181*, *SPAG16*) overlapped with 284 QTLs associated with reproduction traits, further demonstrating the importance of these genes for male fertility. Studies showed that *SERPINE2* can modulate murine sperm capacitation [57, 58]. *AGBL4* and *SORCS1* were related to the sperm motility in Holstein-Friesian bulls [59]. *SPAG16* plays an important role in spermatogenesis [60, 61]. In addition, by calculating the V_ST_, 58 CNVRs overlapping 31 different genes were observed, which were differentiated in HSM and PSM groups. Among those genes, some of them were related to the male fertility, such as *ARID4A*, *ALDH8A1*, *SPAG16* and *ARID4A*, a member of ARID gene family, act as a transcriptional coactivator for androgen receptor and retinoblastoma, can regulate the male fertility and function of sertoli cell [62]. A study reported that *ARID4A* was associated with the semen quality of bulls [63]. *ALDH8A1* can synthesize retinoic acid which plays an important role during spermatogenesis [64, 65]. *SPAG16*, plays an important role in spermatogenesis [60, 61]. In addition, the expression level of *SPAG16* was significantly higher in the cattle testis, compared to other organs (http://animal.nwsuaf.edu.cn/code/index.php/RGD). Notably, a differential deletion (chr2:101427468–101,429,883) was detected following the intronic region of *SPAG16* gene, which was mainly observed in bulls of the PSM group. Meantime, half of the HSM bulls showed a loss of heterozygosity. Moreover, the *SPAG16* was also identified as a candidate gene associated with reproduction by *F*_ST_. Therefore, we speculated that *SPAG16* plays a crucial role in the male fertility.

## Conclusions

In the current study, we performed a comprehensive analysis to explore the genetic variations (SNPs and CNVs) in Simmental cattle. We identified a set of candidate genes associated with reproduction, immunity, milk, and muscle development. In addition, we obtained a confidential CNV dataset and sperm-motility-related CNVRs genes for Simmental cattle using the high-coverage next-generation re-sequencing and long read sequencing. We admitted that this CNV dataset we constructed is not fully complete due to the strict filtering standards, and limited sample. In future research, combining various sequencing platforms and improved detection methods, we may obtain an infinitely close to complete dataset.

## Methods

### Sample collection and genomic sequencing

The frozen semen of 30 Simmental cattle were obtained from Gansu Livestock Breeding Center (Gansu Province, China), which can be divided into high sperm motility (HSM) (n = 14) and poor sperm motility (PSM) (n = 16) based on sperm motility. The sperm motility of fresh semen and frozen-thawed semen were calculated using Minitube Sperm Vision for each individual with at least five ejaculations (CASA SpermVision®, Minitube, Germany). The genomic DNA was extracted using a standard phenol-chloroform protocol [66]. High-quality DNA was processed to construct the short-insert (500 bp) genomic libraries on BGISEQ-500 for genome sequencing (BGI Biotech Co. Ltd, Beijing, China). In addition, one out of 30 bulls was randomly selected for single-molecule long-read sequencing by Nanopore PromethION Platform (Nextomics Biosciences Co., Ltd, Wuhan, China).

### Alignments and variant identification

The cleaned reads of 30 bulls were aligned to the latest high quality reference genome (ARS-UCD1.2) [20] using BWA-MEM with default settings [67]. Duplicate reads were filtered using Picard (v2.5.0). The single nucleotide polymorphisms (SNPs) were detected with the Genome Analysis Toolkit (GATK, version 3.8) [68]. All SNPs were filtered using the “VariantFiltration” implemented in GATK with the standards used in the previous studies [69]. In addition, the Nanopore long reads were mapped to the cattle genome (ARS-UCD1.2) using minimap2 with default settings [70].

### Genome-wide selective sweep tests

The positive genomic regions in Simmental bulls were estimated using three statistical methods, including nucleotide diversity (Pi), integrated Haplotype Score (iHS) and composite likelihood ratio (CLR). The Pi was calculated with 50 kb sliding windows and 20 kb steps along the autosomes using the vcftools. The iHS based on the phased genotype data was performed using selscan v1.1, and score was normalized with the norm module with 50 kb windows and 20 kb increments [71]. The CLR was calculated by SweepFinder2 with each 50-kb window across each chromosome [72]. The KOBAS (http://kobas.cbi.pku.edu.cn/) [22] was used to gain a better understanding of the biological functions and involved pathways. The 3D structures of LEPR was predicted using I-TASSER (https://zhanglab.ccmb.med.umich.edu/I-TASSER/)[73], which were visualized using UCSF Chimera [74].

### CNV identification

Sniffles (Version: 1.0.10) was used to detect structure variation (SV) based on the Nanopore long reads with default parameter [75]. SV analysis outputs were filtered with the following three steps: (1) ambiguous breakpoints (flag: IMPRECISE) and low-quality SV were removed; (2) SVs shorter than 50 bp were removed; (3) SVs with less than four supporting reads were removed. Lumpy (v 0.2.13) was performed for each sample to detect the read-pair and split-read profile CNV call set using the lumpyexpress module with default parameters [76]. CNVnator was used to annotate the copy number [77]. The CNVs were identified as the same type by the three methods to ensure confidence. After considering the intersections between the results of Sniffles, LUMPY and CNVnator, only CNVRs supported by at least two animals were kept (Table S1).

### Detection of candidate genes/CNVRs associated with fertility

The *F*_ST_ was used to scan genomic regions with extreme allele frequency differentiation between HSM and PSM groups using the SNP dataset with 50 kb sliding windows and 20 kb steps along the autosomes by vcftools (0.1.16) [78]. A custom script was used to calculate the V_ST_ using the identified CNVR data set [79]. The formula is VST = (VT - VS)/VT, where VT represents the variance apparent among all unrelated individuals, and VS represents the average variance within each population, weighted for population size. The top 2% V_ST_ were considered to have a significant difference in copy number between the fertile group and sub-fertile group.

### Validation of a deletion within SPAG16 by PCR

The deletion at *SPAG16* (chr2:101,427,468 − 101,429,883) was verified with primers F: 5’-CATGAGGATCAGTGCTGCTG-3’ and R: 5’-GGCACTTCCTTGATCCACACA − 3’. The polymerase chain reaction (PCR) system contained 12.5 µL of 2× EasyTaq PCR SuperMix Polymerase (TransGen Biotech, Beijing, China), 50 ng of genomic DNA, 1 µL of each primer (0.1 µmol/µL), and then adding distilled water to a total volume of 25 µL. The amplification conditions were pre-denaturation at 94 °C for 5 min, followed by 34 cycles of denaturation at 94 °C for 30 s, annealing for 30 s, 72 °C extension for 30 s, and a final extension at 72 °C for 5 min. The PCR products were detected by gel electrophoresis and visualized under UV illumination (Ge1Doc-It TS Imaging System, Upland, CA, USA).

### Functional annotation

In order to explore the biological function and pathway of the identified positive selected genes and CNVR genes, the Kyoto Encyclopedia of Genes and Genomes (KEGG) pathway and Gene Ontology (GO) were performed using KOBAS with a significant threshold for corrected P-value < 0.05 [22]. The cattle quantitative trait loci (QTLs) data were obtained from Animal QTLdb [21]. All QTL data were filtered with *P* ≤ 0.05. To further explore the hereditary effects, the positive selected genes and CNVRs were compared with cattle QTLs using Bedtools with the parameters: -a -b -r -wa -wb [80].

## Electronic supplementary material

Below is the link to the electronic supplementary material.

Supplementary Material 1

Supplementary Material 2

## Data Availability

The raw data have been deposited to NCBI with the BioProject accession number PRJNA701080 and PRJNA950231.

## Declarations

This study was carried out in compliance with the ARRIVE guidelines.

## Abbreviations

BGV: Bovine genome variation database and selective signatures
CGH: Comparative genomic hybridization
CLR: Composite likelihood ratio
CNV: Copy number variation
CNVR: CNV region, YBP:Years before present
GATK: Genome Analysis Toolkit
GWAS: Genome-wide association study
HSM: High sperm motility
iHS: Integrated haplotype Score
Pi: Nucleotide diversity
PSG: Positive selected genes
PSM: Poor sperm motility
QTLs: Quantitative trait loci
RP: Read pair, SR:Split read
RD: Read depth
SNP: Single nucleotide polymorphisms
SV: Structure variation
