# Supplementary material for: Whole genome sequencing of simmental cattle for SNP and CNV discovery
Source: BMC Genomics. 2023 Apr 5;24:179. doi: 10.1186/s12864-023-09248-x (PMC10077681; doi:10.1186/s12864-023-09248-x)
Supplement: Supplementary file 2 — Supplementary Material 2 [file 12864_2023_9248_MOESM2_ESM.docx]

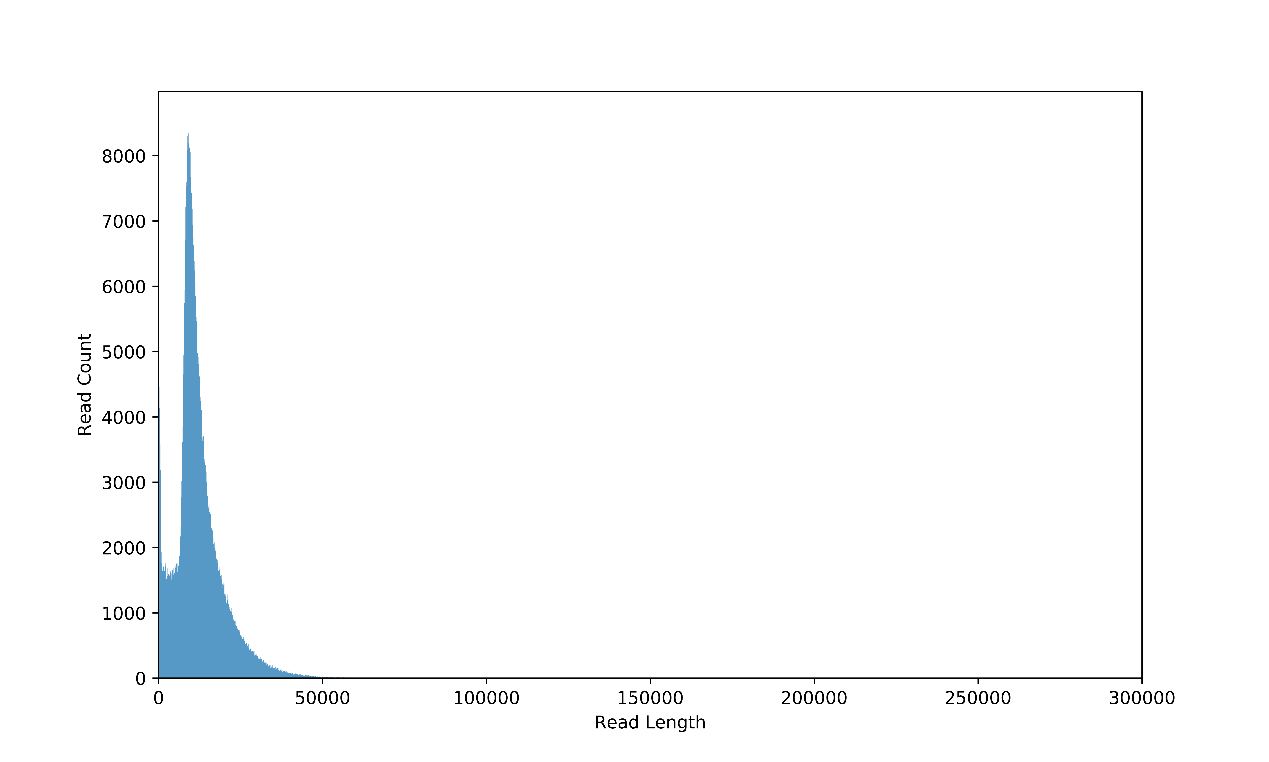


Figure S1 The distribution of read lengths.


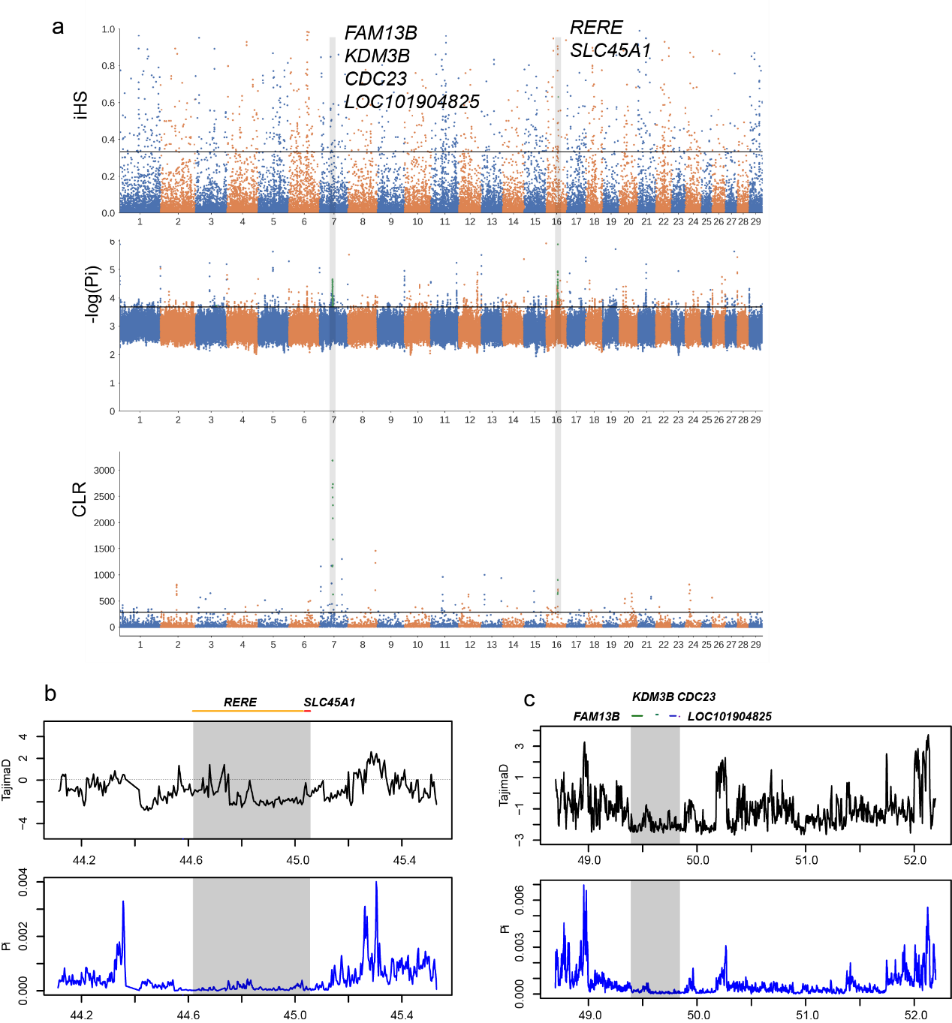


Figure S2 (a) Manhattan plot of selective sweeps in Simental cattle. (b &c ) Selective sweep detected by Tajima’s D and Pi test.


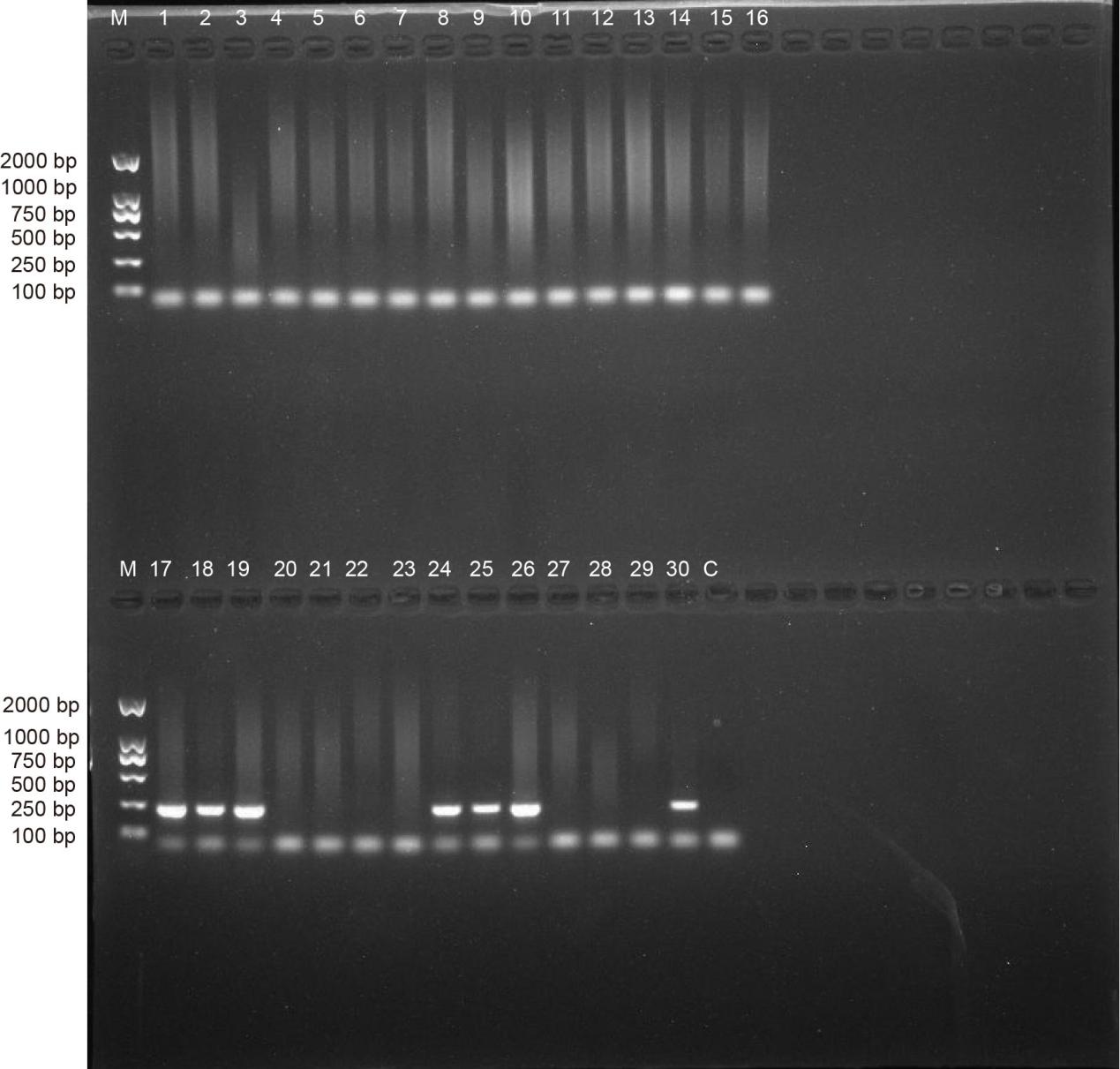


Figure S3 Gel electrophoresis of PCR products of the *SPAG16.* Marker, D2000 DNA ladder, the “M” represents the marker; the “C” represents the blank control (distilled water); 1-16 represents the individuals from PSM group, 17-30 represent individuals from HSM group.
